# Safety and immunogenicity of intramuscularly administered CS6 subunit vaccine with a modified heat-labile enterotoxin from enterotoxigenic *Escherichia coli*

**DOI:** 10.1016/j.vaccine.2021.08.032

**Published:** 2021-09-15

**Authors:** Tida Lee, Ramiro L. Gutiérrez, Milton Maciel, Steven Poole, Kayla J. Testa, Stefanie Trop, Christopher Duplessis, Alison Lane, Mark S. Riddle, Melinda Hamer, Ashley Alcala, Michael Prouty, Nicole Maier, Rahsan Erdem, A. Louis Bourgeois, Chad K. Porter

**Affiliations:** a Enteric Diseases Department, Naval Medical Research Center, United States; b Henry M. Jackson Foundation, United States; c Walter Reed Army Institute of Research, United States; d Uniformed Services University, United States; e PATH, United States

**Keywords:** Enterotoxigenic Escherichia coli, Double mutant heat-labile enterotoxin, ETEC, dmLT, Vaccine, Intramuscular, CS6, CssBA

## Abstract

**Introduction:**

Enterotoxigenic *Escherichia coli* (ETEC) is a common cause of infectious diarrhoea and a leading cause of morbidity and mortality in children living in resource-limited settings. It is also the leading cause of travellers’ diarrhoea among civilian and military travellers. Its dual importance in global public health and travel medicine highlights the need for an effective vaccine. ETEC express colonization factors (CFs) that mediate adherence to the small intestine. An epidemiologically prevalent CF is coli surface antigen 6 (CS6). We assessed the safety and immunogenicity of a CS6-targeted candidate vaccine, CssBA, co-administered intramuscularly with the double-mutant heat-labile enterotoxin, dmLT [LT(R192G/L211A)].

**Methods:**

This was an open-label trial. Fifty subjects received three intramuscular injections (Days 1, 22 and 43) of CssBA alone (5 µg), dmLT alone (0.1 µg) or CssBA (5, 15, 45 µg) + dmLT (0.1 and 0.5 µg). Subjects were actively monitored for adverse events for 28 days following the third vaccination. Antibody responses (IgG and IgA) were characterized in the serum and from lymphocyte supernatants (ALS) to CS6 and the native ETEC heat labile enterotoxin, LT.

**Results:**

Across all dose cohorts, the vaccine was safe and well-tolerated with no vaccine-related severe or serious adverse events. Among vaccine-related adverse events, a majority (98%) were mild with 79% being short-lived vaccine site reactions. Robust antibody responses were induced in a dose-dependent manner with a clear dmLT adjuvant effect. Response rates in subjects receiving 45 µg CssBA and 0.5 µg dmLT ranged from 50 to 100% across assays.

**Conclusion:**

This is the first study to demonstrate the safety and immunogenicity of CssBA and/or dmLT administered intramuscularly. Co-administration of the two components induced robust immune responses to CS6 and LT, paving the way for future studies to evaluate the efficacy of this vaccine target and development of a multivalent, subunit ETEC vaccine.

## 1 Introduction

Enterotoxigenic *Escherichia coli* (ETEC), one of several pathotypes of diarrhoeagenic *E. coli*, causes a secretory diarrhoea that can range in presentation from mild discomfort to cholera-like purging [1], [2]. ETEC is one of the most common causes of childhood diarrhoea in low-and middle-income countries (LMIC), and the estimated number of ETEC-attributable deaths varies from 23,000 to 42,000 (with large uncertainty intervals) annually among infants and young children [3]. In addition to the morbidity associated with acute diarrhoeal illness, recent data indicate that ETEC infections may also be associated with growth faltering and delayed cognitive development [4], [5], further magnifying the pathogen-specific acute and longer-term morbidity and its negative economic impact [6]. ETEC is also the leading cause of travellers' diarrhoea, implicated in 30–50% of cases [7], [8], [9].

While antibiotics have traditionally been effective at clearing ETEC infections, strains have become increasingly resistant [1], [10], [11]. *Enterobacteriaceae* are included on the critical World Health Organisation (WHO) pathogen priority list for the development of new antimicrobials [12] and classified as an urgent antimicrobial resistance (AMR) threat by the US Centers for Disease Control and Prevention [13]. Combined, these data highlight the need for primary prevention. This is supported by the recommendations from the Wellcome Trust and the Boston Consulting Group, as well as the WHO to accelerate vaccine development for enteric *E. coli*, including ETEC [14], [15]. In addition, ETEC vaccine development was also recently reaffirmed as a WHO priority [16].

While ETEC vaccine development has made significant advancements over the past several decades, no vaccine is currently available [17]. To fill this important gap in public health and travel medicine, the US Naval Medical Research Center (NMRC) has been advancing a subunit candidate vaccine based on subunits of common colonization factors (CFs), surface-exposed polymeric protein appendages that mediate initial adherence and colonization of the small intestine. Antibodies directed to CFs have been shown to be protective in natural and experimental infections [18], [19], [20], [21], [22], [23], [24]. One of the most prevalent and epidemiologically important CFs is an atypical polymeric antigen termed CS6 [22], [25], [26]. CS6 is a heteropolymer composed of two subunits, CssA and CssB, in an approximate 1:1 ratio [27]. CS6 binds to Caco-2, INT407 and HT-29 cells [28], [29], [30]. Both purified CS6 and recombinant CssB fused to glutathione-S-transferase (GST) have been shown to bind to intestinal glycosphingolipid sulfatide by thin layer chromatography [31]. CS6-expressing ETEC strains are important causes of diarrhoeal illness among infants and young children in low resource settings and travellers to ETEC-endemic areas [16], [32], [33], [34]. Consequently, it is considered to be an essential component in most ETEC vaccine candidates currently under development [16], [17].

We previously reported on biochemical properties of *in cis* donor strand complemented variants of CssA and CssB and characterized the immune responses in mice [30]. Based on multiple lines of evidence, ntd_14_dsc_16B_CssBA (hereafter termed CssBA), a CssB-CssA fusion in which the N-terminal 14 amino acids of CssB have been removed and a heterologous CssB-derived donor strand is used to complement the C-terminal CssA, was selected as the lead vaccine prototype. Additionally, intramuscular immunization with a formulation containing the CssBA fusion, constructed with the CssA and CssB alleles from ETEC strain B7A, significantly protected *Aotus nancymaee* non-human primates against diarrhoeal disease after challenge with the CS6-expressing ETEC strain B7A [35]. This product was produced under current Good Manufacturing Practice (cGMP) and as detailed here, evaluated for safety and immunogenicity when administered intramuscularly with the double mutant *E. coli* heat-labile enterotoxin LT(R192G/L211A) (dmLT) in a Phase 1 clinical trial. The role of dmLT in the vaccine formulation was to induce anti-LT toxin immunity and to adjuvant anti-CssBA responses [36], [37]. This Phase 1 trial was also the first clinical study in which dmLT was included in a candidate vaccine given by the intramuscular route.

## 2 Methods

### 2.1 Study design

CssBA (a protein fusion of the CssB and CssA subunits of CS6 in which the N-terminal 14 amino acids have been removed from CssB and a sixteen amino acid, heterologous CssB-derived donor strand is used to complement the C-terminal CssA) was selected as the lead vaccine candidate after the biochemical and immunological characterization of a panel of *in cis* donor strand complemented (dsc) variants of CS6 subunit-derived antigens [30]. The source of the CssA and CssB alleles for constructing CssBA was ETEC strain B7A [35]. The vaccine was manufactured at the Walter Reed Army Institute of Research (WRAIR) Pilot Bioproduction Facility (PBF) (Silver Spring, MD) as lot 1880.

Given the likely importance of anti-LT responses in an ETEC vaccine and due to its adjuvant properties, CssBA was co-administered with dmLT (manufacturer: IDT Biologika Corporation; Rockville, MD; Lot: 001 08 16). Currently, there is no previous published human experience of dmLT given by the intramuscular route; however, several first in human studies have been initiated evaluating dmLT intradermally (NCT02531685, NCT01644565).

This study was designed as an open-label, Phase 1 clinical trial in which a total of 50 subjects were scheduled to receive three intramuscular (IM) injections of either CssBA alone, dmLT alone or CssBA + dmLT, with the combined products then dose-escalated by cohort. The vaccine was administered to alternating deltoid regions on Days 1, 22, and 43, and each subject received the same dose at each vaccination dependent upon cohort assignment. Cohort A was considered a pilot group where five subjects received all three vaccinations with either 5 µg CssBA (A-1) or 0.1 µg dmLT (A-2). Subjects in Cohort A were monitored for safety seven days after the third vaccination, before enrollment of subjects in Cohort B. Cohort B consisted of ten subjects receiving 5 µg CssBA dose co-administered with 0.1 µg of dmLT. Following completion of the 3-dose series, the dmLT dose increased to 0.5 µg and co-administered with 5 µg CssBA in Cohort C. The dose of CssBA was then scheduled to be increased to 15 µg (Cohort D) and 45 µg (Cohort E) in subsequent cohorts with either 0.1 or 0.5 µg of dmLT dependent on the safety profile observed with co-administration with 5 µg CssBA. Given no safety concerns in cohort C, subsequent cohorts D and E received the 0.5 µg dmLT dose. Each subject received three doses and all subjects within a cohort were enrolled on the same day.

Healthy adult (aged 18–45 years) male and non-pregnant female subjects were recruited from the greater Washington, DC area through the use of multiple IRB-approved media advertising formats. Interested subjects contacted the WRAIR Clinical Trials Center and discussed details of the trial with a member of the recruitment staff, following which an appointment for briefing/screening was arranged. Subjects were enrolled following completion of an informed consent process which included a taped or in-person presentation about the study, passing a comprehension test, a one-on-one discussion with a clinical investigator and signing the informed consent document. Subjects with any of the following were excluded: significant acute or chronic diseases, immunosuppressive disorders or medication, regular use of anti-diarrhoeal, anti-constipation, or antacid therapy, an abnormal stool pattern (<3 stools per week, or >3 stools per day), participating in other investigational product research within 30 days before the planned date of first vaccination or anytime through the last study safety visit, positive blood test for Hepatitis B surface antigen, Hepatitis C virus, or Human Immunodeficiency Virus-1/2, or clinically significant abnormalities on basic laboratory screening. Subjects with abnormal skin history or findings potentially affecting local adverse event assessments were also excluded. To increase the likelihood of vaccinating immunologically naïve subjects, only those with no history (in the past three years) of ETEC or cholera exposure and with no travel (in the past three years) to countries where those pathogens are endemic were eligible.

The two components were co-formulated for administration immediately before IM vaccination. The vaccine (0.25 ml) was delivered to alternating deltoids using a 23 gauge one-inch needle. Subjects were observed in the clinic for at least 30 min post-vaccination and vital signs were collected. Memory aids were provided to subjects to facilitate adverse event reporting. Subjects returned for follow-up one and seven days after the first vaccination for a clinical evaluation which included vital signs, adverse event assessment, and review of changes in medical history, concomitant medications, and targeted clinical assessment. For subsequent vaccinations, each subject underwent these same clinical evaluations at Days 2 and 8 post-vaccination. Clinical investigators reviewed the memory aid with subjects at each clinical visit. Approximately 6 and 12 months from the last vaccination subjects were contacted by telephone to assess for a final safety assessment.

The decision to advance to the next cohort was based solely on the safety assessment. A dose level with no occurrence of stopping criteria prompted moving to the next higher level. All safety data were summarized and reviewed with the Safety Review Committee (SRC) prior to advancing to dose escalation. Approximately one week after Cohort A (A-1 and A-2) received the third vaccination dose (Day 50), an interim Safety Report was prepared by the PI and Study Statistician for review by the SRC. The content of the report included all adverse events as well as relevant safety endpoints. The SRC’s concurrence to advance to the next cohort was made and provided in written format.

### 2.2 Safety assessment

Safety monitoring was undertaken using in-person symptom surveillance, symptom memory aids, and targeted physical exams. Adverse event (AE) monitoring surveyed and specifically inquired about fever (oral temperature > 100.4°F), malaise, headache, rash, pain, and extremity pain, swelling, or local reactions. Clinical definitions were used to grade the severity of symptoms as mild (not interfering with routine activities), moderate (interfering with but not precluding routine activities) and severe (preventing routine activities). Blood for complete blood counts and serum chemistry was collected throughout the vaccination phase.

### 2.3 Immunological assessments

Immunogenicity measures were assessed throughout the study. Serum samples were assayed for anti-CS6 and –LT antibody (Ab) IgG and IgA titers by enzyme-linked immunosorbent assay (ELISA). Antigen (Ag)-specific IgG assays were performed on Nunc™ MicroWell™, while IgA assays were performed on Nunc™ MicroWell™ Maxisorb™ (Thermo Scientific, Rochester, NY) 96-well plates. For anti-CS6-specific assays, plates were coated with CS6 (BEI, NIH Repository) at 1 or 2 µg/mL (IgG and IgA, respectively), while plates for LT-specific assays were first coated with GM1 (Sigma-Aldrich, Saint Louis, MO) at 0.5 µg/mL, both with 100 µL/well, for 1 h at 37 °C, followed by overnight (O/N) at 4 °C. Plates were blocked with 150 μL/well of 5% non-fat milk (Sigma-Aldrich) in 0.05% Tween-20 (Sigma-Aldrich)-PBS (PBS-T) for CS6 assays, or 1% Casein (Sigma-Aldrich) for LT assays, for 60 min at 37 °C in a humidified chamber. LT (courtesy Prof. Elizabeth Norton, Tulane University) was added to LT-specific plates at 0.2 µg/mL and incubated at 37 °C for 1 h. After three washes with PBS-T, serum samples were added at a starting dilution of 1:50 in 1% or 2% non-fat milk-PBS-T (for CS6 IgG and IgA assays, respectively), or 0.5% Casein (for LT IgG and IgA assays), followed by a 3-fold serial dilution, and incubated for 1.5 h at 37 °C in a humidified chamber. Plates were washed 5 times with PBS-T followed by addition of 0.25 µg/mL biotin-conjugated anti-human IgG or IgA (KPL), for CS6 IgG and IgA, and LT IgA assays, or 0.5 µg/mL peroxidase-conjugated goat anti-human IgG (KPL, Gaithersburg, MD) for LT IgG assays, for 1.5 h at room temperature (RT). After further washes, assays performed with biotin-conjugates received ExtrAvidin®-Peroxidase (Sigma-Aldrich) at 1:2000 for 30 min at RT. After final washes, 2,2′-azino-bis(3-ethylbenzothiazoline-6-sulphonic acid (ABTS; KPL) substrate was used to develop assays based on peroxidase, while 3,3′,5,5′-tetramethylbenzidine (Ultra-TMB; Thermo Scientific) substrate was employed to develop assays based on biotin-strepavidin, according to the manufacture recommendations. After a 20–30 min incubation, optical density (OD) was measured at 450 or 405 nm for ABTS or Ultra-TMB, respectively, using a Multiskan EX® ELISA reader with Ascent® software (Thermo Scientific), which calculated the final antibody titers. The cut-off for each plate was calculated by the average of the background wells OD plus a fixed value of 0.4. A linear regression was fitted to the experimental data and the endpoint titer was determined as the reciprocal of the interpolated sample dilution that intersected with the cut-off and then log_10_-transformed. All pre- and post-vaccination samples from a given subject were assayed concurrently on the same plate and each sample was tested in duplicate. The average log_10_ reciprocal titer for the duplicate tests was calculated as the final result. Serum samples with OD below the cut-off, even at the top serum dilution, were assigned a value of one-half of the lower detection limit for analysis.

After isolation by a Ficoll gradient with Cell Preparation Tubes with sodium heparin (CPT™; Becton, Dickson and Company, Franklin Lakes, NJ, US), peripheral blood mononuclear cells were cultured at 5x10^6^ cells/mL in 24-well plates (Becton, Dickson and Company, Falcon^TM^) in compete RPMI (10% FCS, 1% Penicillin/Streptomicin, 1% GlutMax) without stimulation for 72 h. Once collected, supernatants were kept at −80 °C until assayed by ELISA for anti-CS6 and anti-LT IgG and IgA antibody as described above, except for the initial sample dilution, which were performed as follows: 1:8 for anti-CS6 IgG, 1:5 for anti-CS6 IgA, and 1:5 for anti-LT IgG and IgA. A positive response for serology and antibody from lymphocyte supernatant (ALS) was defined as a four-fold rise in antibody titers between pre- and post-vaccination samples.

### 2.4 Statistical analysis

Given the early stage of the product concept/testing, the sample size for this study was designed to evaluate preliminary safety data but not designed to show statistically significant differences between cohorts. Nominal data (proportion with adverse events, proportion meeting immunologic responder definitions) were analyzed by Pearson’s Chi-square test (or Fisher’s exact test) to compare dose levels. For immune responses, antibody titers were log_10_-transformed for analysis. Between cohorts, comparisons were examined using analysis of variance (ANOVA) and over time using repeated measures ANOVA. Only subjects who received at least two vaccine doses were included in immunologic analysis. All analyses were performed using SAS v 9.4 (Cary, NC) and GraphPad Prism 7.0 for Mac (GraphPad Software, La Jolla, CA) and a two-sided alpha = 0.05.

## 3 Results

### 3.1 Safety

**Fig. 1:**
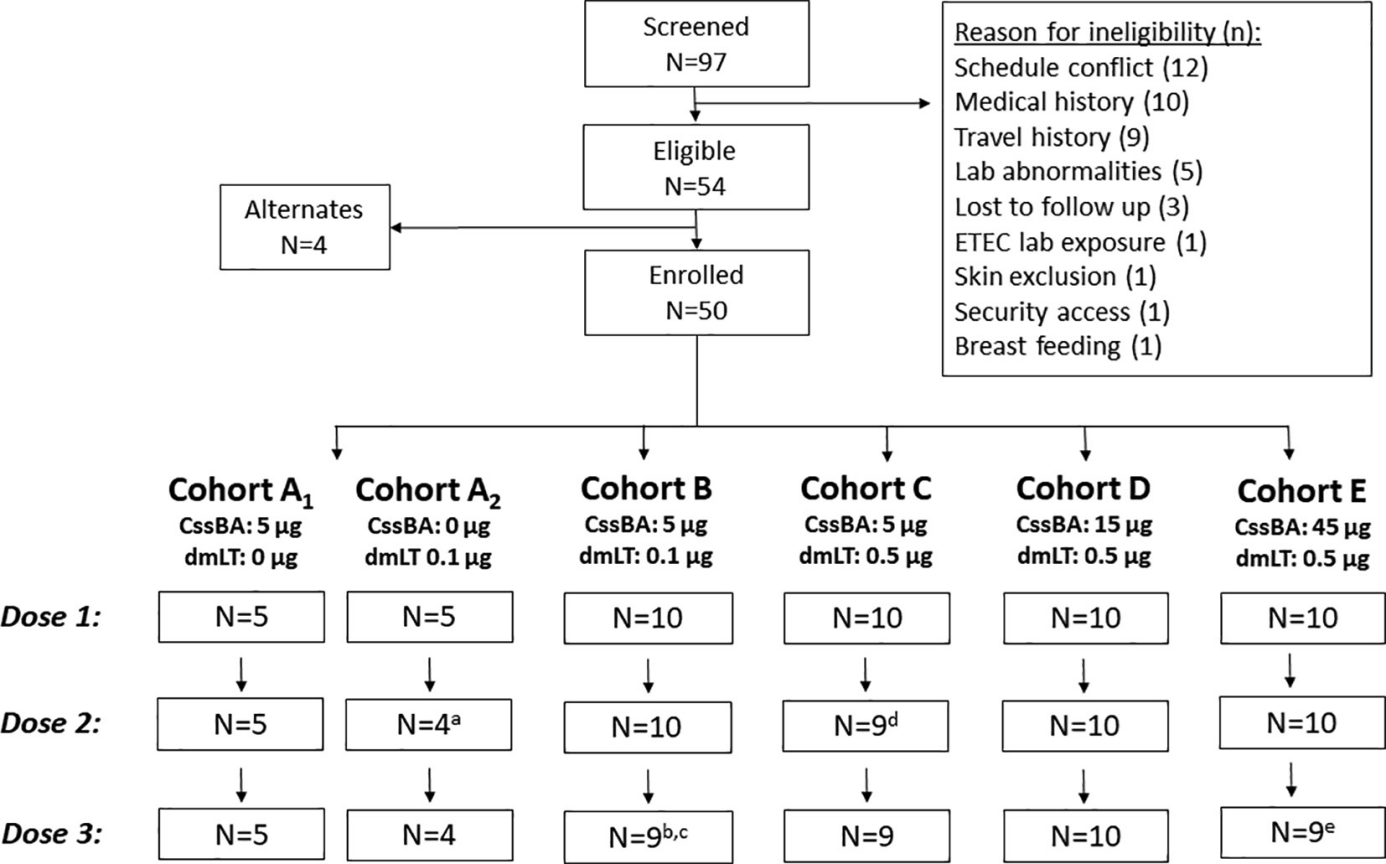
Study population diagram. a. Subject withdrawn after first dose due to a schedule change leading to inability to attend future visits; b. subject withdrawn after two doses due to schedule changes that prevented the subject from attending future visits; c. one subject received three doses but was withdrawn after the third dose due to behavioral issues; d. Subject withdrawn after first dose due to initiation of a medication that precluded eligibility; e. Subject was withdrawn after two doses due to initiation of a medication that precluded eligibility.

A total of 97 subjects were screened to identify 50 eligible subjects (Fig. 1). The most common reasons for ineligibility were schedule conflict (12/43), medical history (10/43), and history of travel to an ETEC endemic area (9/43). Among the 54 eligible subjects, 50 were ultimately enrolled in the study with a median age of 30 years (interquartile range, IQR: 26–37). Of the included population, 60% were female, 46% were black and 48% were white, and the majority were non-Hispanic (Table 1). Five subjects were withdrawn from the study, two subjects after one dose, two subjects after two doses, and one subject was withdrawn after three doses. None of the subject withdrawals were due to AEs but were due to either an inability to comply with the study procedures or starting medication that precluded the subjects’ continued eligibility.

**Table 1:** Demographics of study population.

	A-1	A-2	B	C	D	E	Total
**N**	5	5	10	10	10	10	50
**Median Age (1Q, 3Q)**	32 (30, 41)	29 (27, 37)	29.5 (26, 32)	31.5 (27, 37)	28 (22, 38)	29.5 (25, 33)	30 (26, 37)
**Sex**							
Male	1 (20.0)	3 (60.0)	5 (50.0)	3 (30.0)	5 (50.0)	3 (30.0)	20 (40.0)
Female	4 (80.0)	2 (40.0)	5 (50.0)	7 (70.0)	5 (50.0)	7 (70.0)	30 (60.0)

**Race**							
Asian	0 (0.0)	0 (0.0)	0 (0.0)	2 (20.0)	0 (0.0)	0 (0.0)	2 (4.0)
Black	4 (80.0)	4 (80.0)	3 (30.0)	5 (50.0)	3 (30.0)	4 (40.0)	23 (46.0)
White	1 (20.0)	1 (20.0)	7 (70.0)	3 (30.0)	7 (70.0)	5 (50.0)	24 (48.0)
Multi-racial	0 (0.0)	0 (0.0)	0 (0.0)	0 (0.0)	0 (0.0)	1 (10.0)	1 (2.0)

**Ethnicity**							
Non-Hispanic/Latino	5 (100.0)	3 (60.0)	9 (90.0)	10 (100.0)	8 (80.0)	9 (90.0)	44 (88.0)
Hispanic/Latino	0 (0.0)	2 (40.0)	1 (10.0)	0 (0.0)	2 (20.0)	1 (10.0)	6 (12.0)

Across all study cohorts, the vaccine was safe and well-tolerated. The frequency of vaccine-related systemic AEs differed slightly across cohorts, with the lowest frequency in subjects receiving CssBA alone (Table 2). Loose or soft stools and headache at least possibly related to the vaccine occurred in less than 20% of subjects in all cohorts. Transient myalgia was observed in 10.0–20.0% of subjects receiving 0.5 μg dmLT and was not observed among subjects receiving CssBA alone or 0.1 μg dmLT; however, this difference was not statistically significant (p = 0.5).

**Table 2:** Systemic adverse events considered to be at least possibly related to the vaccine.

Adverse Event	Cohort A-1 (N = 5)	Cohort A-2 (N = 5)	Cohort B (N = 10)	Cohort C (N = 10)	Cohort D (N = 10)	Cohort E (N = 10)	Total (N = 50)
Abdominal Pain	0 (0.0)	0 (0.0)	0 (0.0)	0 (0.0)	1 (10.0)	0 (0.0)	1 (2.0)
Arthralgia	0 (0.0)	0 (0.0)	0 (0.0)	0 (0.0)	1 (10.0)	1 (10.0)	2 (4.0)
Aspartate Aminotransferase Increased	0 (0.0)	1 (20.0)	0 (0.0)	0 (0.0)	0 (0.0)	0 (0.0)	1 (2.0)
Chills	0 (0.0)	0 (0.0)	0 (0.0)	0 (0.0)	1 (10.0)	0 (0.0)	1 (2.0)
Loose/Soft stools	0 (0.0)	1 (20.0)	2 (20.0)	2 (20.0)	2 (20.0)	1 (10.0)	8 (16.0)
Fatigue	0 (0.0)	0 (0.0)	1 (10.0)	0 (0.0)	0 (0.0)	0 (0.0)	1 (2.0)
Headache	1 (20.0)	0 (0.0)	1 (10.0)	1 (10.0)	2 (20.0)	0 (0.0)	5 (10.0)
Leukopenia	0 (0.0)	0 (0.0)	0 (0.0)	0 (0.0)	1 (10.0)	0 (0.0)	1 (2.0)
Malaise	0 (0.0)	0 (0.0)	0 (0.0)	0 (0.0)	2 (20.0)	1 (10.0)	3 (6.0)
Myalgia	0 (0.0)	0 (0.0)	0 (0.0)	1 (10.0)	2 (20.0)	2 (20.0)	5 (10.0)
Nausea	0 (0.0)	0 (0.0)	0 (0.0)	1 (10.0)	1 (10.0)	0 (0.0)	2 (4.0)
Pruritus, inner arm	0 (0.0)	0 (0.0)	0 (0.0)	0 (0.0)	0 (0.0)	1 (10.0)	1 (2.0)
Pyrexia	0 (0.0)	0 (0.0)	0 (0.0)	0 (0.0)	1 (10.0)	0 (0.0)	1 (2.0)

Adverse events at the vaccine site were mostly mild (98%) with erythema and tenderness being the most common reactions across cohorts (Table 3). Erythema occurred in the majority of subjects receiving dmLT (70% of all subjects; A-2: 80.0%; B: 70.0%; C: 70.0%; D: 80.0%; E: 80.0%). Vaccine site pain was more prevalent among subjects in Cohorts C (50.0%), D (80.0%), and E (80.0%) compared to Cohorts A (10.0%) and B (20.0%) (Fisher’s exact, p = 0.003). Additionally, induration was only observed in subjects receiving the 0.5 μg of dmLT (p = 0.002) and there appeared to be a CssBA-driven dose response effect on the timing of the induration. Specifically, 6 (66.7%) subjects in Cohort C (5 μg CssBA, 0.5 μg dmLT) experienced induration only after the third dose. In Cohorts D (15 μg CssBA, 0.5 μg dmLT) and E (45 μg CssBA, 0.5 μg dmLT), induration increased with each dose (Cohort D- dose 1: 0.0%, dose 2: 30.0%, dose 3: 50.0%; Cohort E- dose 1: 20.0%, dose 2: 40.0%, dose 3: 66.7%). Erythema and pain were seen more frequently after the third dose among subjects in all cohorts (p = 0.013 and p = 0.002, respectively). In Cohort D, 2 (20.0%) and 6 (60.0%) subjects had pruritus after the second vaccination and third vaccinations, respectively, and only two subjects (one each in Cohorts B and C) had pruritus (p < 0.0001).

**Table 3:** Vaccine-site reactions by dose.

		Vaccine Site Solicited Symptom and Severity [N (%)]														
		Pain			Pruritus			Erythema			Swelling			Induration		
Cohort	Dose	Mild	Mod	Sev	Mild	Mod	Sev	Mild	Mod	Sev	Mild	Mod	Sev	Mild	Mod	Sev
A-1	1 (n = 5)	0 (0.0)	0 (0.0)	0 (0.0)	0 (0.0)	0 (0.0)	0 (0.0)	0 (0.0)	0 (0.0)	0 (0.0)	0 (0.0)	0 (0.0)	0 (0.0)	0 (0.0)	0 (0.0)	0 (0.0)
	2 (n = 5)	0 (0.0)	0 (0.0)	0 (0.0)	0 (0.0)	0 (0.0)	0 (0.0)	1 (20.0)	0 (0.0)	0 (0.0)	0 (0.0)	0 (0.0)	0 (0.0)	0 (0.0)	0 (0.0)	0 (0.0)
	3 (n = 5)	0 (0.0)	0 (0.0)	0 (0.0)	0 (0.0)	0 (0.0)	0 (0.0)	0 (0.0)	0 (0.0)	0 (0.0)	0 (0.0)	0 (0.0)	0 (0.0)	0 (0.0)	0 (0.0)	0 (0.0)
A-2	1 (n = 5)	1 (20.0)	0 (0.0)	0 (0.0)	0 (0.0)	0 (0.0)	0 (0.0)	1 (20.0)	0 (0.0)	0 (0.0)	0 (0.0)	0 (0.0)	0 (0.0)	0 (0.0)	0 (0.0)	0 (0.0)
	2 (n = 4)	1 (25.0)	0 (0.0)	0 (0.0)	0 (0.0)	0 (0.0)	0 (0.0)	1 (20.0)	0 (0.0)	0 (0.0)	0 (0.0)	0 (0.0)	0 (0.0)	0 (0.0)	0 (0.0)	0 (0.0)
	3 (n = 4)	0 (0.0)	0 (0.0)	0 (0.0)	0 (0.0)	0 (0.0)	0 (0.0)	4 (100.0)	0 (0.0)	0 (0.0)	0 (0.0)	0 (0.0)	0 (0.0)	0 (0.0)	0 (0.0)	0 (0.0)
B	1 (n = 10)	1 (10.0)	0 (0.0)	0 (0.0)	1 (10.0)	0 (0.0)	0 (0.0)	4 (40.0)	0 (0.0)	0 (0.0)	0 (0.0)	0 (0.0)	0 (0.0)	0 (0.0)	0 (0.0)	0 (0.0)
	2 (n = 10)	1 (10.0)	0 (0.0)	0 (0.0)	1 (10.0)	0 (0.0)	0 (0.0)	2 (20.0)	0 (0.0)	0 (0.0)	0 (0.0)	0 (0.0)	0 (0.0)	0 (0.0)	0 (0.0)	0 (0.0)
	3 (n = 9)	0 (0.0)	0 (0.0)	0 (0.0)	0 (0.0)	0 (0.0)	0 (0.0)	3 (33.3)	0 (0.0)	0 (0.0)	0 (0.0)	0 (0.0)	0 (0.0)	0 (0.0)	0 (0.0)	0 (0.0)
C	1 (n = 10)	4 (40.0)	0 (0.0)	0 (0.0)	0 (0.0)	0 (0.0)	0 (0.0)	4 (40.0)	0 (0.0)	0 (0.0)	0 (0.0)	0 (0.0)	0 (0.0)	0 (0.0)	0 (0.0)	0 (0.0)
	2 (n = 9)	2 (22.2)	0 (0.0)	0 (0.0)	0 (0.0)	0 (0.0)	0 (0.0)	6 (66.7)	0 (0.0)	0 (0.0)	1 (11.1)	0 (0.0)	0 (0.0)	0 (0.0)	0 (0.0)	0 (0.0)
	3 (n = 9)	2 (22.2)	0 (0.0)	0 (0.0)	1 (11.1)	0 (0.0)	0 (0.0)	6 (66.7)	0 (0.0)	0 (0.0)	0 (0.0)	0 (0.0)	0 (0.0)	6 (66.7)	0 (0.0)	0 (0.0)
D	1 (n = 10)	6 (60.0)	0 (0.0)	0 (0.0)	0 (0.0)	0 (0.0)	0 (0.0)	0 (0.0)	0 (0.0)	0 (0.0)	0 (0.0)	0 (0.0)	0 (0.0)	0 (0.0)	0 (0.0)	0 (0.0)
	2 (n = 10)	4 (40.0)	0 (0.0)	0 (0.0)	2 (20.0)	0 (0.0)	0 (0.0)	5 (50.0)	0 (0.0)	0 (0.0)	0 (0.0)	0 (0.0)	0 (0.0)	3 (30.0)	0 (0.0)	0 (0.0)
	3 (n = 10)	6 (60.0)	1 (10.0)	0 (0.0)	6 (60.0)	0 (0.0)	0 (0.0)	8 (80.0)	0 (0.0)	0 (0.0)	1 (10.0)	0 (0.0)	0 (0.0)	5 (50.0)	0 (0.0)	0 (0.0)
E	1 (n = 10)	2 (20.0)	0 (0.0)	0 (0.0)	0 (0.0)	0 (0.0)	0 (0.0)	1 (10.0)	0 (0.0)	0 (0.0)	0 (0.0)	0 (0.0)	0 (0.0)	2 (20.0)	0 (0.0)	0 (0.0)
	2 (n = 10)	5 (50.0)	1 (10.0)	0 (0.0)	0 (0.0)	0 (0.0)	0 (0.0)	7 (70.0)	0 (0.0)	0 (0.0)	0 (0.0)	0 (0.0)	0 (0.0)	4 (40.0)	0 (0.0)	0 (0.0)
	3 (n = 9)	4 (44.4)	0 (0.0)	0 (0.0)	0 (0.0)	0 (0.0)	0 (0.0)	6 (66.7)	0 (0.0)	0 (0.0)	0 (0.0)	0 (0.0)	0 (0.0)	6 (66.7)	0 (0.0)	0 (0.0)

Two subjects (1 in Cohort B and 1 in Cohort C) had bruising at the vaccination site.

One subject (Cohort E) had vaccine-site discoloration and a papule at the vaccination site after second and third vaccinations.

Adverse event severity was characterized as mild: not interfering with routine activities; moderate (mod): interfering with but not precluding routine activities; severe (sev): preventing routine activities.

### 3.2 Immunogenicity

**Fig. 2:**
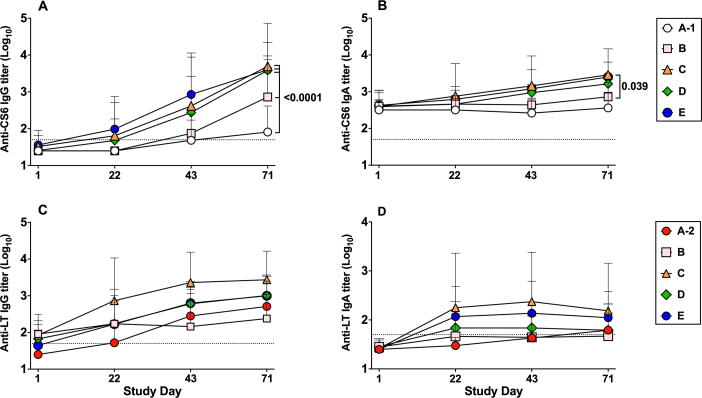
Kinetics of serum antigen-specific antibody responses. **(A)** Anti-CS6 IgG titers; **(B)** Anti-CS6 IgA titers; **(C)** Anti-LT IgG titers; **(D)** Anti-LT IgA titers. Results are shows as geometric means ± geometric SD. Dotted horizontal line indicates the limit of detection of the assay. The peaks of responses of each cohort were compared by ANOVA followed by Tukey post-hoc comparisons and considered significantly different when p < 0.05.

**Fig. 3:**
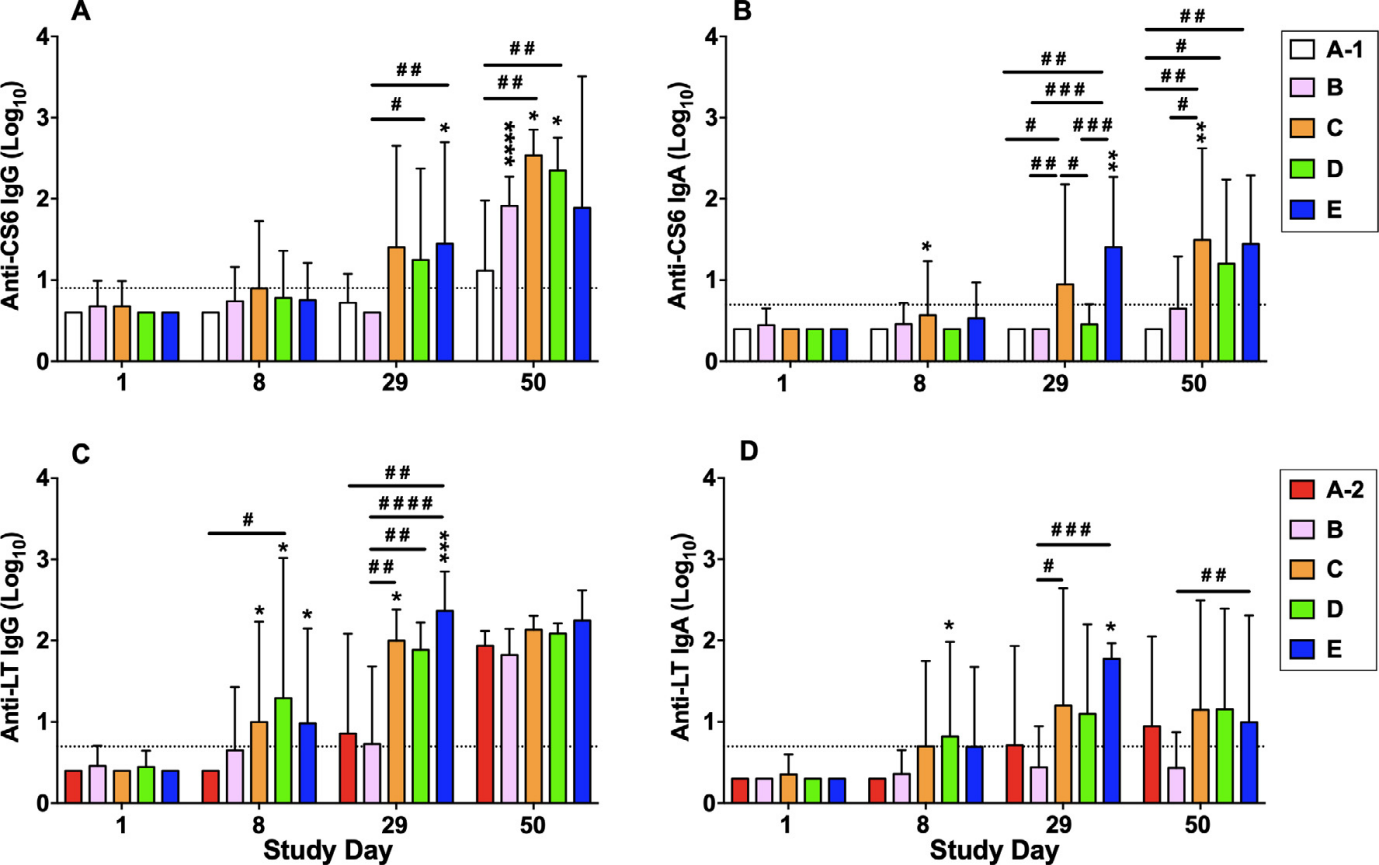
Kinetics of ALS antigen-specific responses. **(A)** Anti-CS6 IgG; **(B)** Anti-CS6 IgA; **(C)** Anti-LT IgG; **(D)** Anti-LT IgA. Results are shows as geometric mean ± geometric SD. Dotted horizontal line indicates the limit of detection of the assay. Statistical comparisons between time points of the same cohort were performed ANOVA for repeated measures and indicated as *p < 0.05, **p < 0.01, ***p < 0.001, and ****p < 0.0001; Statistical comparisons between cohorts for a given time point were performed by ANOVA followed by Tukey *ad hoc* test, and indicated as ^#^p < 0.05, ^# #^p < 0.01, ^# # #^p < 0.001, and ^# # # #^p < 0.0001.

The frequency of anti-CS6 serologic responses in subjects receiving 5 μg CssBA alone (Group A-1) was low across all assays (Table 4). When 5 μg CssBA was co-administered with 0.1 μg of dmLT (Group B), the anti-CS6 IgA response rate remained low; however, serum IgG responses increased to 77.8% (7/9). Increasing the dmLT dose to 0.5 μg (Group C) induced a higher proportion of serum IgG and IgA responders (100% (9/9) and 44.4% (4/9), respectively). Anti-CS6 serum IgG and IgA antibody levels increased over the study period with peak titers observed on Day 71 for subjects receiving CssBA + dmLT (Fig. 2**A and B**). Anti-CS6 IgG titers were significantly higher in subjects receiving 5 μg CssBA + 0.1 μg dmLT compared to those receiving 5 μg CssBA alone (p = 0.0001). Increasing the dmLT dose to 0.5 μg dmLT in the subsequent cohorts (C, D and E) further increased the CS6 IgG titers in comparison to cohort A-1 (p < 0.0001) (Fig. 2**A**). A significant increase in peak anti-CS6 IgA antibody titers was also observed between groups B and C when the dmLT dose was increased from 0.1 to 0.5 μg (p = 0.04, Fig. 2**B**). ALS anti-CS6 response rate increased with the addition of 0.1 μg dmLT (IgG: 60% (3/5) to 100% (8/8); IgA: 0% (0/5) to 33% (3/9)) (Table 4). Increasing the dose of dmLT to 0.5 μg induced >80% ALS response rates in all subsequent groups regardless of CssBA dose. Overall, the ALS titers peaked after the third immunization, on Day 50 (Fig. 3**A and B**). Among all groups, vaccination with 5 µg CssBA + 0.5 µg dmLT (Group C) elicited the highest mean titers; however, those titers were not significantly different than those observed in subjects receiving higher doses of CssBA with 0.5 µg dmLT.

**Table 4:** Proportion of Subjects Demonstrating an Immunologic Response (Serology, ALS) to Immunizing Antigens.

		Study Cohort [n (%)]					
Antigen	Assay	A-1 5 µg CssBA (N = 5)	A-2 0.1 µg dmLT (N = 4)	B 5 µg CssBA + 0.1 µg dmLT (N = 9)^a^	C 5 µg CssBA+ 0.5 µg dmLT (N = 9)	D 15 µg CssBA+ 0.5 µg dmLT (N = 10) (N = 10)	E 45 µg CssBA+ 0.5 µg dmLT (N = 10)
Coli Surface Antigen 6 (CS6)	Serology (IgA)	0 (0.0)	0 (0.0)	0 (0.0)	4 (44.4)	3 (30.0)	5 (50.0)
	Serology (IgG)	1 (20.0)	0 (0.0)	7 (77.8)	9 (100.0)	10 (100.0)	9 (90.0)
	ALS (IgA)	0 (0.0)	0 (0.0)	3 (33.3)	8 (88.9)	8 (80.0)	10 (100.0)
	ALS (IgG)	3 (60.0)	0 (0.0)	8^b^ (100.0)	8 (88.9)	10 (100.0)	8 (80.0)
Heat labile toxin (LT)	Serology (IgA)	0 (0.0)	2 (50.0)	2 (22.2)	7 (77.8)	5 (50.0)	7 (70.0)
	Serology (IgG)	0 (0.0)	4 (100.0)	6 (66.7)	9 (100.0)	9 (90.0)	10 (100.0)
	ALS (IgA)	0 (0.0)	4 (100.0)	2 (22.2)	7 (77.8)	9 (90.0)	10 (100.0)
	ALS (IgG)	0 (0.0)	4 (100.0)	8^b^ (100.0)	9 (100.0)	10 (100.0)	10 (100.0)

Responder defined as ≥ 4-fold rise in baseline reciprocal endpoint titer.

a One subject excluded because the subject only had one sample post vaccination despite receiving two vaccinations.

b One subject excluded because the subject only had one ALS sample post vaccination despite receiving two vaccinations.

Anti-LT serum antibody responses were frequent with significant increases over baseline titers for all groups receiving dmLT (Table 4**;** Fig. 2**C and 2D**). Serum anti-LT IgG antibody titers appeared to increase over the vaccination series (Fig. 2**C**), while anti-LT IgA titers peaked after first or second vaccination, depending on the group (Fig. 2**D).** All groups receiving dmLT had a significant increase in ALS anti-LT IgG and IgA levels (Table 4; Fig. 3**C and D**). ALS anti-LT IgG titers increased significantly after the first vaccination for Groups C, D, and E (p < 0.05 for all comparisons) and continued to increase significantly after the second vaccination in Groups C and E (p < 0.01 and p < 0.001, respectively) (Fig. 3**C**). By Day 50, seven days after the third vaccination, all groups had comparable ALS anti-LT IgG titers. Compared to baseline, ALS anti-LT IgA antibody titers only increased significantly in group D after the first vaccination, by Day 8 (p < 0.05; Fig. 3**D**). Significant increases in ALS anti-LT IgA levels were also seen in Group E after the second vaccination, by Day 29 (p < 0.05).

Supplementary data associated with this article can be found, in the online version, at https://doi.org/10.1016/j.vaccine.2021.08.032.

**Suppl. Fig. 1:**
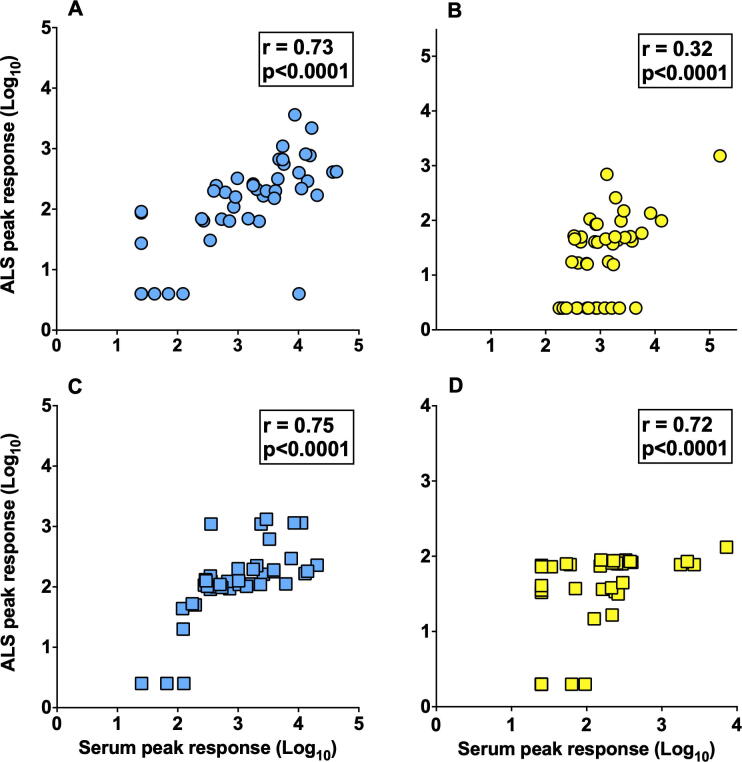
Correlation between serum and ALS antigen-specific peak responses. (A) Anti-CS6 IgG; (B) Anti-CS6 IgA; (C) Anti-LT IgG; (D) Anti-LT IgA. Correlations between serum and ALS peak responses in all cohorts (n=45) were analyzed by Spearman and considered significantly different when p<0.05.

Strong correlations were observed between serological and ALS responses for anti-CS6 IgG, and anti-LT IgG and IgA antibodies (all Spearman r = 0.72 to 0.75, p < 0.0001; Suppl. Fig. 1A, C and D), while a modest correlation was observed for the anti-CS6 IgA antibody response (Spearman r = 0.32, p < 0001; Suppl. Fig. 1B).

## 4 Discussion

This is the first publication on the safety and immunogenicity of the CssBA subunit candidate vaccine and the first evaluation of dmLT administered IM. The investigational products were well-tolerated at all doses and the majority (97.0%) of vaccine-related AEs were considered mild. The most common symptom related to vaccination was erythema at the site of administration, observed in 70% of vaccine recipients. Vaccination site pain was also common (48%) and local site reactions seemed most prevalent in subjects receiving dmLT at the 0.5 μg dose. Others have evaluated dmLT alone or as an adjuvant through oral, sublingual, and intradermal (ID) routes, without safety concerns [38], [39] (NCT02531685). For the ID route, the highest dose of dmLT tested to date is 2.0 µg with no aberrant safety concerns. Previously, we observed that mice immunized ID with 2.5 µg of dmLT developed local induration, that was limited and self-resolving [37], and IM delivery of up to 1.0 µg in BALB/c mice dose induced some local swelling that spontaneously resolved in 14–21 days (M. Maciel, unpublished observations). These data, combined with the positive safety profile of the doses administered in this study, indicate that it may be possible to use a higher dose of dmLT in future clinical trials.

In addition to being well-tolerated, IM immunization with CssBA + dmLT induced robust systemic serum IgG and IgA responses, similar to our preclinical observations [36], [37]. Additionally, as measured by ALS as proxy to antibody-secreting cells, vaccination induced robust activation of the B cell compartment against CS6 and LT in a dose-dependent manner, with the most robust responders observed in groups receiving CssBA with the 0.5 µg dmLT dose.

Together, these data suggest that recombinant CssBA co-administered IM with a potent adjuvant such as dmLT is able to induce a robust immune response against the native antigen CS6. Additionally, dmLT also induced a robust anti-LT response. While anti-LT titers significantly increased following the first vaccination among subjects receiving the 0.5 µg dmLT dose, overall anti-CS6 responses tended to increase in magnitude and frequency with increasing CssBA doses. There were no significant differences in the response rates or the peak responses in subjects receiving 15 and 45 µg of CssBA (with 0.5 μg dmLT). Further analyses are being performed with faecal and saliva samples, as well as with mucosal-homing α4β7^+^ antibody-secreting cells, to assess whether the IM vaccination with CssBA + dmLT is able to elicit mucosal responses as has been seen with other parenterally administered vaccines [40]. These data will be important given that mucosal antibody production is thought to be required for protection against ETEC-attributable illness [17].

These results support the ongoing development of a subunit-based vaccine strategy based on the intramuscular administration of subunits of common CFs along with dmLT. In addition to CssBA, the currently envisioned final vaccine formulation includes CfaEB, CsbDA-CooA, and CotDA. CfaEB, a fusion of the adhesin (CfaE) and pilin (CfaB) subunits of CFA/I, is anticipated to provide coverage against ETEC strains expressing class 5a fimbriae (CFA/I, CS4, and CS14) and is a second generation form of the CfaE antigen that has demonstrated efficacy in *Aotus nancymaae* and safety and immunogenicity in humans (NCT01382095; NCT01644565; NCT01922856) [41], [42]. CsbDA-CooA, a pilin-adhesin fusion containing the adhesin (CsbD) and the pilin (CsbA) of CS17 and the pilin of CS1, is anticipated to cover all class 5b fimbriae ETEC strains (CS1, CS17, CS19, and PCFO71). Lastly, CotDA, a fusion of the adhesin (CotD) and pilin (CotA) of CS2, would provide coverage against strains expressing CS2 fimbriae (class 5c). Such a vaccine, potentially co-administered with other subunit antigens, would also be co-administered with dmLT. Preclinical investigations of the multivalent formulation in small rodents demonstrated that each vaccine component elicited functional antibodies against its respective CF (M. Maciel, personal communication). Data indicate that this quadrivalent vaccine would provide coverage of the majority of ETEC strains causing disease in adult travelers and children living in LMICs [32], [33].

This subunit-based vaccine approach is amenable to the characteristics delineated in the WHO’s recently published draft preferred product characteristics for an ETEC vaccine [16]. Additionally, given that this vaccine is administered intramuscularly, it may offer an advantage over orally administered vaccines which have traditionally been met with reduced immunogenicity when administered to infants and young children in endemic settings [43], [44], [45]. Furthermore, its ability to be combined with other enteric vaccines under development, including *Shigella* and typhoid, may increase the value of the ETEC subunits vaccine approach [16], [43], [44], [46].

## 5 Disclaimer

The views expressed in this article are those of the authors and do not necessarily reflect the official policy or position of the Department of the Navy, Department of the Army, Department of Defense, nor the U.S. Government. This is a US Government work. There are no restrictions on its use. There were no financial conflicts of interests among any of the authors.

## 6 Copyright statement

Some authors are employees of the U.S. Government. This work was prepared as part of their official duties. Title 17 U.S.C. §105 provides that “Copyright protection under this title is not available for any work of the United States Government.” Title 17 U.S.C. §101 defines a U.S. Government work as a work prepared by a military service member or employee of the U.S. Government as part of that person’s official duties.

## Funding

This study was funded by 10.13039/100005624PATH under a Cooperative Research and Development Agreement (NMR 9589). This work was supported, in part, by the 10.13039/100000865Bill & Melinda Gates Foundation (OPP1112376). Under the grant conditions of the Foundation, a Creative Commons Attribution 4.0 Generic License has already been assigned to the Author Accepted Manuscript version that might arise from this submission.

## Declaration of Competing Interest

The authors declare that they have no known competing financial interests or personal relationships that could have appeared to influence the work reported in this paper.
